# Lipid goal attainment in post‐acute coronary syndrome patients in China: Results from the 6‐month real‐world dyslipidemia international study II

**DOI:** 10.1002/clc.23725

**Published:** 2021-10-15

**Authors:** Yanjun Gong, Xuan Li, Xiang Ma, Hongwei Yu, Ying Li, Jiyan Chen, Guochun Zhang, Bin Wang, Xiaoyong Qi, Haiyan Meng, Xiaofeng Wang, Jianjun Mu, Xitian Hu, Jingping Wang, Shaowen Liu, Gang Liu, Zhenyu Yang, Yujie Zhou, Xiangqing Kong, Yuhu Yan, Changqian Wang, Jian' An Wang, Lijun Wang, Guosheng Fu, Lin Wei, Daoquan Peng, Shuyang Zhang, Ruogu Li, Anhua Mao, Rui Bian, Wenmin Tang, Yuqin Ran, Jie Jiang, Yong Huo

**Affiliations:** ^1^ Department of Cardiology Peking University First Hospital Beijing China; ^2^ Department of Cardiology Jinqiu Hospital of Liaoning Province Shenyang China; ^3^ Department of Cardiology The First Affiliated Hospital of Xinjiang Medical University Urumqi China; ^4^ Department of Cardiology Zhengzhou First People's Hospital Zhengzhou China; ^5^ Department of Cardiology Shanghai East Hospital Shanghai China; ^6^ Department of Cardiology Guangdong Provincial People's Hospital Guangzhou China; ^7^ Department of Cardiology The Second Hospital of Harbin Harbin China; ^8^ Department of Cardiology The First Affiliated Hospital of Shantou University Medical College Shantou China; ^9^ Department of Cardiology Hebei General Hospital Shijiazhuang China; ^10^ Department of Rehabilitation Shandong Provincial Third Hospital Jinan China; ^11^ Department of Cardiology Traditional Chinese Medicine Hospital of Xinjiang Uygur Autonomous Region Urumqi China; ^12^ Department of Cardiology The First Affiliated Hospital of Xi'an Jiaotong University Xi'an China; ^13^ Department of Cardiology Shijiazhuang First Hospital Shijiazhuang China; ^14^ Department of Cardiology Cardiovascular Hospital of Shanxi Province Taiyuan China; ^15^ Department of Cardiology Shanghai General Hospital Shanghai China; ^16^ Department of Cardiology The First Hospital of Hebei Medical University Shijiazhuang China; ^17^ Department of Cardiology Wuxi People's Hospital Wuxi China; ^18^ Department of Cardiology Beijing Anzhen Hospital, Capital Medical University Beijing China; ^19^ Department of Cardiology Jiangsu Province Hospital Nanjing China; ^20^ Department of Cardiology Jinzhong First People's Hospital Jinzhong China; ^21^ Department of Cardiology The Ninth People's Hospital, Shanghai Jiaotong University School of Medicine Shanghai China; ^22^ Department of Cardiology The Second Affiliated Hospital of Zhejiang University School of Medicine Hangzhou China; ^23^ Department of Cardiology Shijiazhuang the Third Hospital Shijiazhuang China; ^24^ Department of Cardiology Sir Run Shaw Hospital, Zhejiang University School of Medicine Hangzhou China; ^25^ Department of Cardiology The First Hospital of Harbin Harbin China; ^26^ Department of Cardiology The Second Xiangya Hospital of Central South University Changsha China; ^27^ Department of Cardiology Peking Union Medical College Hospital Beijing China; ^28^ Department of Cardiology Shanghai Chest Hospital Shanghai China; ^29^ Global Medical and Scientific Affairs (GMSA) MSD China Shanghai China

**Keywords:** acute coronary syndrome, DYSIS II, LDL‐C goal, lipid‐lowering therapy, statin

## Abstract

**Background:**

Dyslipidemia International Study II (DYSIS II)‐China was conducted to determine the low‐density lipoprotein cholesterol (LDL‐C) goal (<1.8 mmol/L) attainment rate in patients with post‐acute coronary syndrome (ACS).

**Hypothesis:**

Compliance with treatment guideline recommendations improves the LDL‐C goal attainment rate in post‐ACS patients.

**Methods:**

This multicenter prospective observational study conducted at 28 tertiary hospitals determined the LDL‐C goal attainment rates at admission and 6‐month follow‐up in patients on lipid‐lowering treatment (LLT) for ≥3 months and those not on LLT (LLT‐naive or off LLT for ≥3 months) at admission. Predictors of goal attainment at 6 months were identified using multivariate logistic regression.

**Results:**

The LDL‐C goal attainment rate at admission in 1102/1103 enrolled patients was 17.1%; it was 41.2% among 752 patients with available lipid results at 6 months. The distance to goal was 0.7 mmol/L at 6 months. Statin monotherapy was the most prescribed LLT. Only 7.7% of patients were receiving statin + ezetimibe and 8.4% of patients were receiving an atorvastatin‐equivalent dose of ≥40 mg/day at 6 months. Being male (odds ratio [OR] 1.7, 95% confidence interval [CI] 1.1–2.6) and undergoing percutaneous coronary intervention during index hospitalization (OR 1.5, 95% CI 1.1 to 2.1) were the independent predictors for LDL‐C goal attainment.

**Conclusions:**

This real‐world DYSIS II study in China reports a low LDL‐C goal attainment rate in post‐ACS patients even after 6 months of LLT. Lack of intensification of statin therapy and underutilization of combinations suggest gaps between real‐world treatment practices and guideline recommendations.

## 1 INTRODUCTION

Ischemic heart disease (IHD) is one of the leading causes of death in China.^1^ Acute coronary syndrome (ACS) is an acute manifestation of IHD and includes ST elevation myocardial infarction (STEMI), non‐ST elevation myocardial infarction (NSTEMI), and unstable angina (UA).^2^

The introduction of percutaneous coronary intervention (PCI), as well as advanced antithrombotic and antiplatelet treatments, has led to a significant improvement in outcomes in patients presenting with ACS.^3^ However, patients surviving ACS episodes remain at high‐risk for recurrent atherothrombotic events.^4^, ^5^ The GRACE registry reported 6‐month postdischarge death rates of 4.8%, 6.2%, and 3.6% for patients with STEMI, NSTEMI, and UA, respectively.^6^ These mortality rates mandate the need for a rigorous and persistent monitoring with optimum long‐term medical management of patients' post‐ACS to improve their survival. Elevated low‐density lipoprotein cholesterol (LDL‐C) level is a major risk factor for the development and recurrence of ACS.^7^ Lipid‐lowering therapy (LLT) has been identified as a positive predictor of LDL‐C goal attainment.^8^, ^9^ Statin‐based LLT reduces the risk of subsequent cardiovascular (CV) events such as cause‐specific mortality, and major vascular events in patients with stable coronary heart disease (CHD) or ACS.^10^ Intensive LLT in ACS survivors has demonstrated improved long‐term clinical outcomes in PROVE‐IT^11^ and IMPROVE‐IT^12^ studies.

The 2016 Chinese guidelines for the management of dyslipidemia in adults adopted the 2011 European Society of Cardiology (ESC) and the European Atherosclerosis Society (EAS) guidelines,^13^ recommending intensive lipid control with an LDL‐C goal <1.8 mmol/L (<70 mg/dl) for patients with very high‐risk CHD (atherosclerotic CV disease, including ACS). For people who cannot achieve this target level, a reduction in LDL‐C level by at least 50% is recommended. The guidelines recommend the use of medium‐intensity statins for the initial treatment of patients who present with ACS, with optimal dose‐titration to achieve target lipid levels. A combination of statins with other LLT is recommended for patients who do not achieve lipid goals.^14^ In 2019, ESC/EAS lowered the LDL‐C goal to <1.4 mmol/L (<55 mg/dl) with an LDL‐C reduction of ≥50% from baseline in very high‐risk patients for secondary prevention. In case of nonachievement of the goal in 4–6 weeks, the highest tolerated statin dose and ezetimibe is recommended; a PCSK9 inhibitor is also recommended to be added.^15^

There is a lack of evidence to understand the gaps between guideline recommendations and real‐world long‐term treatment practices for secondary prevention (post‐ACS), necessitating to determine the LDL‐C goal attainment rates in China. A recent cross‐sectional study from a region of China based on electronic medical records from 2001 to 2018 reports a low LDL‐C goal attainment (LDL‐C < 1.8 mmol/L) rate of 33.8% in patients having a recent episode of ACS.^16^ ESC/EAS also suggest evidence generation regarding attainment of recommended LDL‐C goals among very high‐risk patients in real‐world practice. Thus we conducted a real‐world observational dyslipidemia international study II (DYSIS II) study to assess LDL‐C goal attainment in a prospective manner in a large pool of patients across multiple centers representing patients with ACS in China. We report on patterns of LLT, and LDL‐C goal attainment at baseline (ACS) and at 6 months post‐ACS in China.

## 2 METHODS

DYSIS II‐China was a multicenter prospective observational study conducted between September 2017 and May 2019 at 28 cardiology departments of tertiary hospitals. The study protocol was approved by the ethics committee of each hospital according to the local regulations and the study was performed in accordance with the Declaration of Helsinki. Each patient provided written informed consent for participation before enrolment. The study included patients hospitalized for an ACS, with the availability of full lipid profile results performed within 24 hours of hospital admission, and were either on LLT ≥3 months (defined as LLT group: prescribed with statins: atorvastatin, fluvastatin, lovastatin, pravastatin, rosuvastatin, simvastatin, pitavastatin and/or nonstatin lipid lowing agents ezetimibe, fibrates, nicotinic acids, laropiprant, omega3) or ‘LLT‐naive or off LLT for ≥3 months’ (defined as non‐LLT group). An atorvastatin‐equivalent dose of other statins was estimated by assuming their equivalent dose for each dose level of atorvastatin. Patients with any cognitive impairment at discharge were excluded. 6‐month follow‐up visits were conducted when patients visited hospitals for their routine clinical care at around Day 180 ± 30 of enrolment. The sample size was calculated to be 96 patients with an assumption of a lower one‐sided precision at 6% for an estimated LDL‐C goal attainment rate of 21% in the smallest subgroup of ‘nonstatin users’ of the study population at a 6‐month follow‐up. Considering approximately 10% of patients to be ‘nonstatin users’ in the total study population and a dropout rate of 15%, the sample size for the overall study was calculated to be 1130.

### 2.1 Data collection/variables

At baseline, the data collection included data on demographic and clinical variables such as age, gender, region, body mass index, type of ACS, sedentary lifestyle, smoking status, and comorbidities such as hypertension, type 2 diabetes mellitus (T2DM), hypercholesterolemia, chronic kidney disease, chronic heart failure (CHF), history of ACS, myocardial infarction (MI), stroke, and coronary revascularization were captured as per history provided by patients/ caregivers and study investigator's/treating physician's assessment. Laboratory data as per local clinical practice at baseline and 6‐month follow‐up visits included lipid profile (serum levels of total cholesterol [TC], LDL‐C, high‐density lipoprotein cholesterol (HDL‐C), non‐HDL‐C, and triglycerides). Baseline high‐sensitivity C‐reactive protein (hsCRP) levels were also recorded. LLT and other concomitant medications at admission, during the hospital stay, discharge, and at 6‐month follow‐up were recorded.

Data on reason(s) for study discontinuation were also recorded during follow‐up visits. The LDL‐C goal of <1.8 mmol/L was considered as the cut‐off for lipid control.^14^ At admission, patients were categorized according to their lifetime estimated CV risk at pre‐event as having very high‐risk, high‐risk, medium, and low risk.^14^ This was based on pre‐ACS event conditions. Rate of LDL‐C goal attainment recommended for patient's risk strata (very high‐risk [LDL‐C < 1.8 mmol/L], high‐risk [LDL‐C < 2.6 mmol/L], medium and low risk [LDL‐C < 3.4 mmol/L]) as per 2016 Chinese guideline was assessed at admission.

### 2.2 Statistics analysis

Point estimates (percentage) for goal attainment rate were calculated with a corresponding two‐sided 95% CI at baseline (admission) and the 6‐month follow‐up visit by Wilson score method for all patients, LLT patients, and non‐LLT patients. The differences in the point estimates (percentage) were compared between the groups using Chi‐square and Wald asymptotic method for a binomial proportion. A multivariate logistic regression model was constructed for identifying the predictors of LDL‐C goal attainment.

## 3 RESULTS

### 3.1 Patient disposition

**FIGURE 1:**
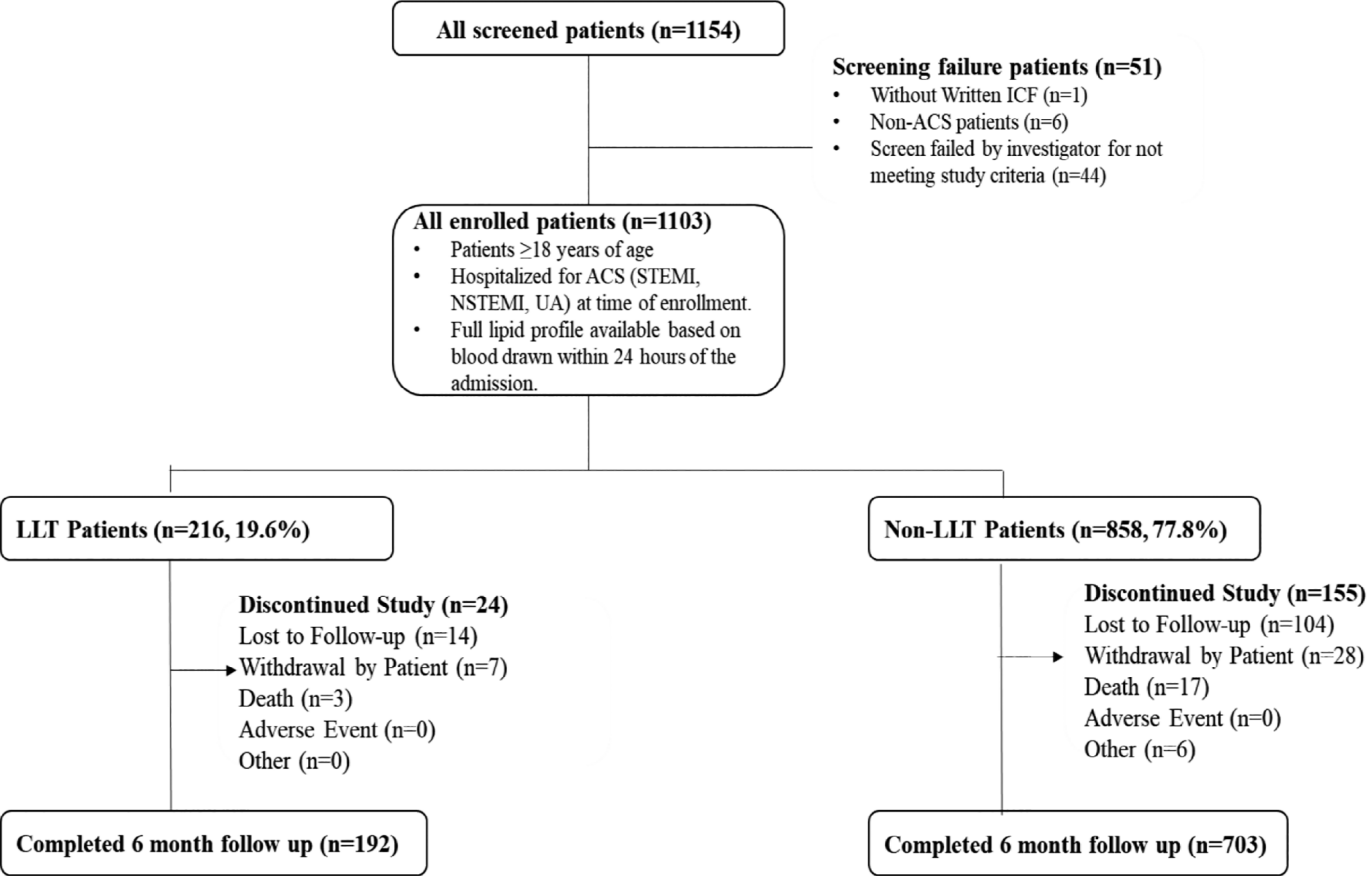
Patient disposition in the study. ACS, acute coronary syndrome; ICF, informed consent form; LLT, lipid‐lowering therapy; NSTEMI, non‐ST elevation myocardial infarction; STEMI, ST elevation myocardial infarction; UA, unstable angina. LLT group: Patients on LLT ≥3 months; Non‐LLT group: LLT‐naive or off LLT for ≥3 months. *All enrolled patients also include 29 patients who had taken some dose of LLT during the 3 months prior to enrolment but could not be categorized in LLT or non‐LLT groups as per protocol‐specified definitions

Of the 1154 patients screened, 1103 patients were enrolled. Of the enrolled patients, those receiving some dose of LLT within 3 months of enrolment (*n* = 29) could not be categorized in the LLT or non‐LLT group; therefore, they were only included in the all patients group. Of the remaining patients, 216 were enrolled in the LLT group and 858 in the non‐LLT group. A total of 192 patients from the LLT group and 703 from the non‐LLT group returned for 6‐month clinic visits (median follow‐up duration: 6.3 months, interquartile range 1.1) and completed study assessments. In all, 907 patients completed the study including patients who could not be categorized as LLT or non‐LLT. Figure 1 describes the patient disposition.

### 3.2 Baseline demographics and clinical profile

The mean age of the study population was 61.7 ± 11.3 years; about 75.0% of the patients were men. Overall, UA (53.2%) was more prevalent followed by STEMI (29.7%) and NSTEMI (17.0%). About 47.7% (521/1092) of the patients were smokers; 35.4% (347/979) had a sedentary lifestyle. The median hsCRP was 3.0 mg/L. At admission, 19.6% (216/1103) of patients were on LLT. Compared to the non‐LLT group, the LLT group had higher percentages of patients with a history of ACS, stroke, MI, symptomatic CHF, UA, hypercholesterolemia, hypertension, T2DM, and coronary revascularization (Table 1).

**TABLE 1:** Demographic and clinical profile

Characteristics/categories	All patients	LLT patients	Non‐LLT patients
Age, mean ± SD (years)^a^	61.7 ± 11.3 (*N* = 1103)	64.9 ± 10.0 (*N* = 216)	60.9 ± 11.4 (*N* = 858)
Gender, male, % (*n*/*N*)	75.0 (827/ 1103)	67.1 (145/216)	76.9 (660/858)
BMI, mean ± SD (kg/m^2^)	24.9 ± 3.3 (*N* = 1074)	25.5 ± 3.3 (*N* = 214)	24.8 ± 3.3 (*N* = 832)
hsCRP, median (range), (mg/L)	3.0 (0.–178.0) (*N* = 667)	1.8 (0.–78.4) (*N* = 119)	3.6 (0.0–178.0) (*N* = 525)
Type of ACS,% (*n*/*N*)			
STEMI	29.7 (328/1103)	6.5 (14 /216)	35.7 (306/858)
NSTEMI	17.0 (188/1103)	10.6 (23/216)	19.1 (164/858)
UA	53.2 (587/1103)	82.9 (179/216)	45.2 (388/858)
History of ACS, % (*n*/*N*)	29.6 (309/1043)	67.3 (138/205)	19.0 (154/810)
History of MI, % (*n*/*N*)	12.2 (131/1072)	29.4 (62/211)	6.8 (57/833)
History of stroke, % (*n*/*N*)	9.6 (100/1045)	14.9 (29/194)	8.1 (67/823)
History of symptomatic CHF (NYHA II‐IV), % *n*/*N*	6.5 (68/1050)	9.8 (20/204)	5.7 (47/818)
History of hypercholesterolemia, % (*n*/*N*)	13.0 (136/1043)	23.6 (47/199)	9.9 (81/816)
History of hypertension, % (*n*/*N*)	62.4 (686/1100)	71.2 (153/215)	59.9 (513/857)
History of T2DM, % (*n*/*N*)	28.6 (313/1096)	38.8 (83/214)	25.5 (218/854)
History of CKD, % (*n*/*N*)	3.0 (32/1055)	4.5 (9/202)	2.8 (23/825)
History of coronary revascularization, % (*n*/*N*)	17.9 (191/1066)	53.1 (112/211)	8.2 (68/828)
Sedentary lifestyle, % (*n*/*N*)	35.4 (347/979)	39.6 (74/187)	33.5 (257/767)
Smoking, % (*n*/*N*)	47.7 (521/1092)	33.5 (72/215)	51.0 (433/849)
Lipid levels, mean ± SD (mmol/L)	(N = 1102)	(N = 216)	(N = 857)
TC	4.3 ± 1.2	3.7 ± 1.0	4.5 ± 1.2
LDL‐C	2.7 ± 0.9	2.2 ± 0.7	2.8 ± 1.0
HDL‐C	1.1 ± 0.3	1.0 ± .0.3	1.1 ± 0.3
Non‐HDL‐C	3.2 ± 1.1	2.7 ± 1.0	3.4 ± 1.1
TG	1.7 ± 1.3	1.7 ± 1.5	1.9 ± 1.3
Pre‐admission CV risk stratification, % (*n*/*N*)			
Very high	43.9 (483/1101)	82.9 (179/216)	33.0 (283/857)
High	28.6 (315/1101)	6.9 (15/216)	34.5 (296/857)
Medium	11.9 (131/1101)	4.6 (10/216)	14.1 (121/857)
Low	15.6 (172/1101)	5.6 (12/216)	18.3 (157/857)

Abbreviations: ACS, acute coronary syndrome; BMI, body mass index; CHF, chronic heart failure; CKD, chronic kidney disease; CV, cardiovascular; HDL‐C, high‐density lipoprotein cholesterol; hsCRP, high‐sensitivity C‐reactive protein; LDL‐C, low‐density lipoprotein cholesterol; LLT, lipid‐lowering therapy; MI, myocardial infarction; NSTEMI, non‐ST elevation myocardial infarction; NYHA, New York Heart Disease Association; SD, standard deviation; STEMI, ST elevation myocardial infarction; T2DM, type 2 diabetes mellitus; TC, total cholesterol; TG, triglycerides; UA, unstable angina.

^a^ Percentages, means, and medians were based on the number of patients with valid data.

The mean levels of lipid parameters were TC 4.3 ± 1.2 mmol/L (LLT 3.7 mmol/L ± 1.0, non‐LLT 4.5 ± 1.2 mmol/L), LDL‐C 2.7 ± 0.9 mmol/L (LLT 2.2 ± 0.7 mmol/L, non‐LLT 2.8 ± 1.0 mmol/L); both levels were lower in the LLT group at baseline.

As per pre‐admission risk assessment, an overall 43.9% (483/1101) of patients were categorized as very high‐CV risk. The majority of the LLT patients (82.9%) were at very high‐risk before admission, whereas the non‐LLT patients were evenly distributed among four risk levels, very high (33.0%), high (34.5%), medium (14.1%), and low (18.3%) (Table 1).

### 3.3 Attainment of lipid goals

**FIGURE 2:**
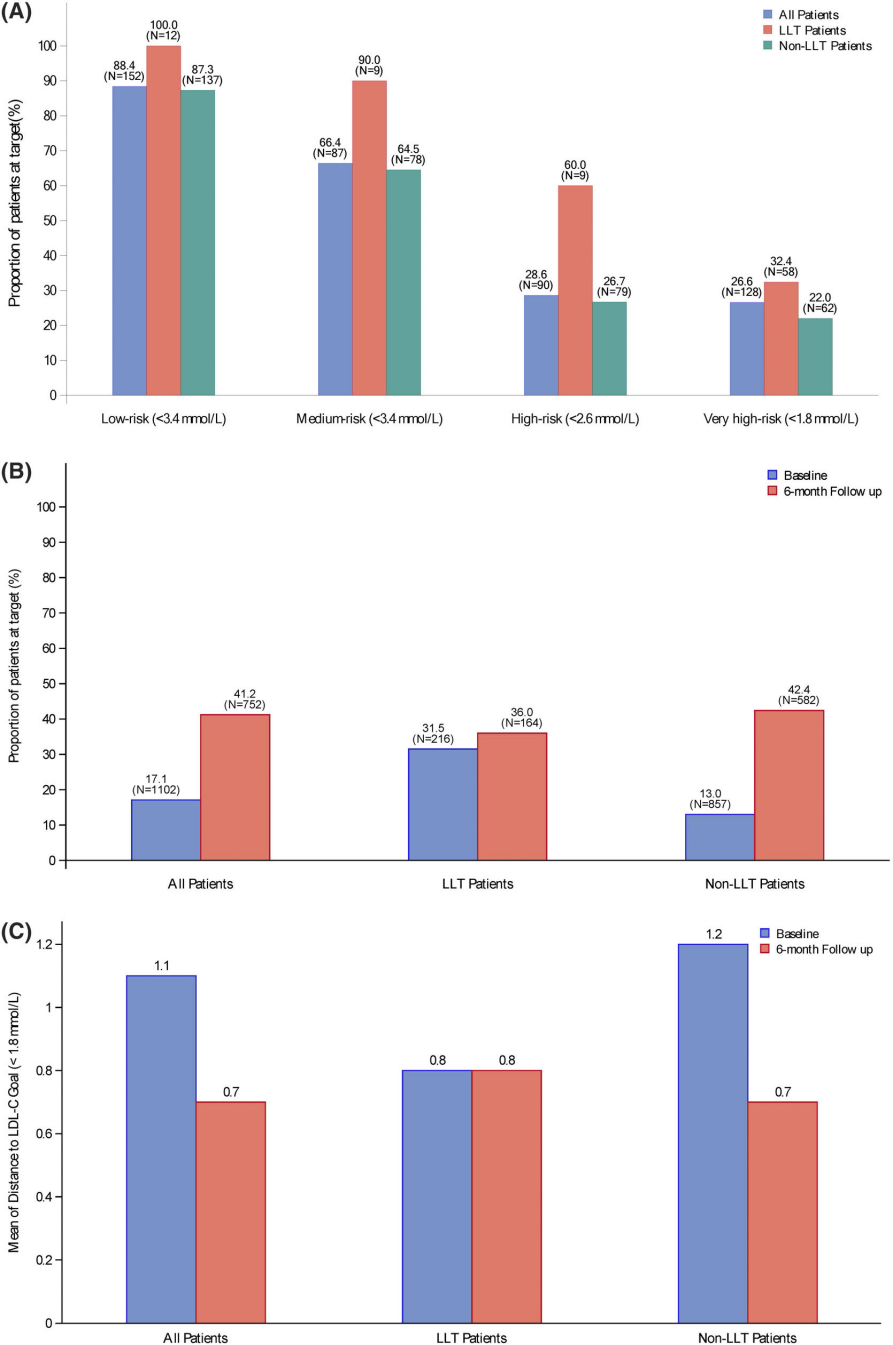
LDL‐C goal attainment rates and distance to LDL‐C Goal. LDL‐C, low‐density lipoprotein cholesterol; LLT, lipid‐lowering therapy. Figure 2 (A) LDL‐C goal attainment rate as per pre‐admission risk classification; Figure 2 (B): LDL‐C goal attainment rate at baseline and 6‐month follow‐up; Figure 2 (C) Distance to LDL‐C Goal at baseline and 6‐month follow‐up

At admission, a higher percentage of patients in the LLT group were at their LDL‐C goal for their category defined by the Chinese guideline, compared with those not on LLT, irrespective of pre‐admission CV risk categories (Figure 2A). Overall, 17.1% (188/1102) of patients at admission (baseline) and 41.2% (310/752 with available lipid profile results) at 6 months had LDL‐C level < 1.8 mmol/L (Figure 2B). Although not significant, at 6‐month follow‐up, a comparatively higher percentage of patients from the non‐LLT group (42.4%), after being started on LLT, achieved the LDL‐C goal than those who were in the LLT group (36.0%, *p* = 0.137) (Figure 2B). The mean LDL‐C level at 6‐month follow‐up for all ACS patients was 2.1 ± 0.8 mmol/L, while for LLT and non‐LLT groups, the levels were 2.2 ± 0.8 mmol/L and 2.1 ± 0.8 mmol/L, respectively. In overall patients who could not attain the LDL‐C goal, the mean distance to LDL‐C goal reduced from 1.1 ± 0.8 mmol/L at baseline to 0.7 ± 0.7 mmol/L at 6‐month follow‐up. The distance to LDL‐C goal in the non‐LLT group reduced from 1.2 ± 0.99 mmol/L at baseline to 0.7 ± 0.7 mmol/L at 6‐month follow‐up (Figure 2(C)).

### 3.4 Lipid‐lowering therapy

**FIGURE 3:**
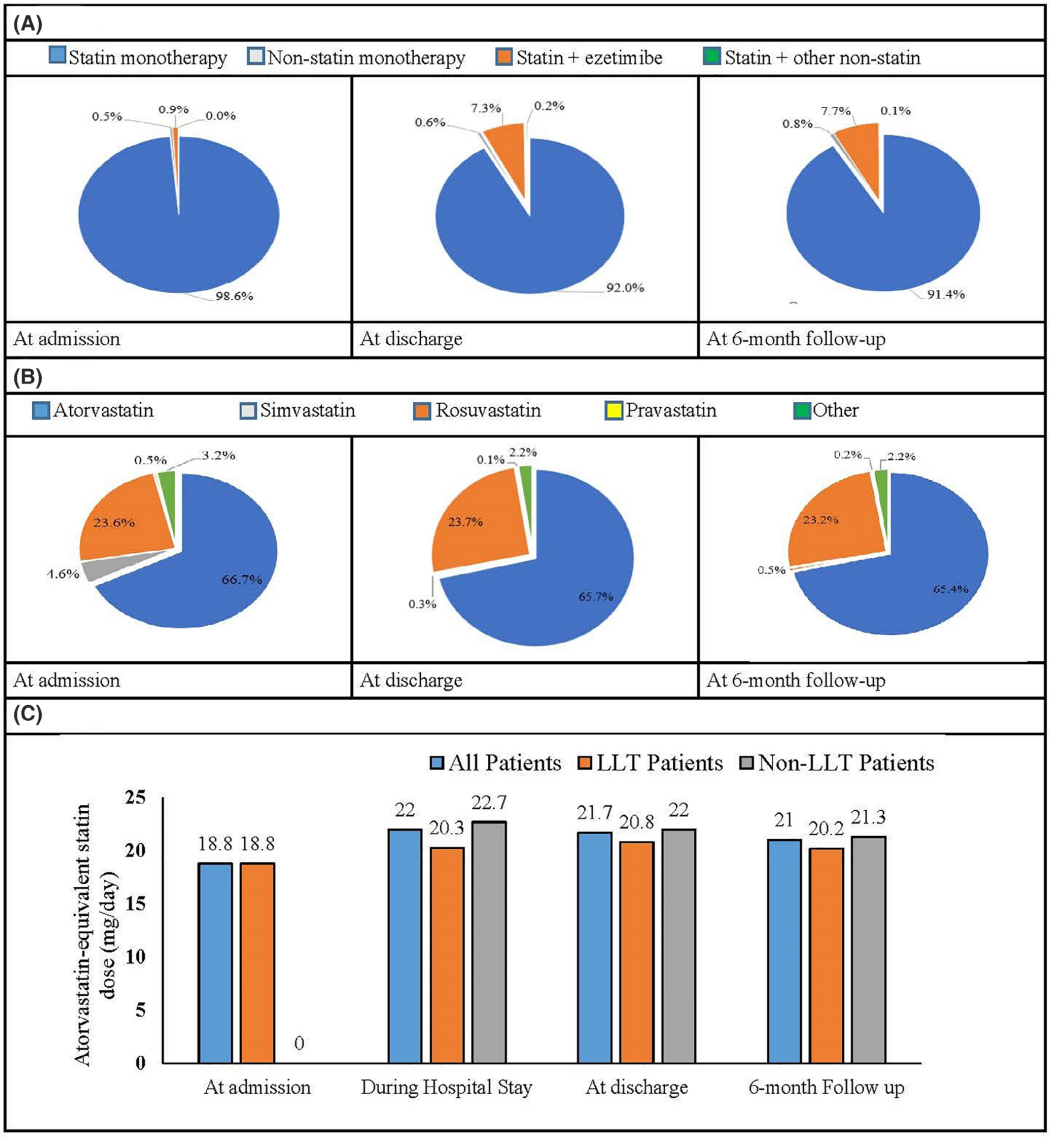
Lipid‐lowering therapy. ACS, acute coronary syndrome; LLT, lipid‐lowering therapy. Figure 3A LLT treatment pattern in all ACS patients at three time‐points: Admission, discharge, and 6‐month follow‐up; Figure 3B: Statin category in all ACS patients at three time‐points: Admission, discharge, and 6‐month follow‐up; Figure 3C: Atorvastatin‐equivalent daily statin dosage at four time‐points in all patients, LLT and Non‐LLT groups. Atorvastatin‐equivalent dose calculation: Atorvastatin 5 mg = simvastatin (10 mg), fluvastatin (40 mg), lovastatin (20 mg), pravastatin (20 mg), pitavastatin (1 mg); Atorvastatin 10 mg = simvastatin (20 mg), fluvastatin (80 mg), lovastatin (40 mg), pravastatin (40 mg), pitavastatin (2–4 mg), rosuvastatin (5 mg); Atorvastatin 20 mg = simvastatin (40 mg), lovastatin (80 mg), pravastatin (80 mg), rosuvastatin (10 mg); Atorvastatin 40 mg = simvastatin (80 mg), rosuvastatin (20 mg); Atorvastatin 80 mg = rosuvastatin (40 mg)

Statin monotherapy was the most frequently used LLT in all ACS patients at all time‐points during the study (Figure 3, Supplementary Table 1). Among the patients who were on LLT at admission, 98.6% received statin monotherapy and 0.9% of patients received statin + ezetimibe. Statin monotherapy was given to most patients at discharge from the hospital and at 6‐month follow‐up (Figure 3A, Supplementary Table 1). Atorvastatin monotherapy was the most commonly used LLT (at admission [LLT group]: 66.7%; at discharge: all patients: 65.7%, LLT group: 60.5%, non‐LLT group: 67.5%; and at 6‐month follow‐up: all patients: 65.4%, LLT group: 59.8%, non‐LLT group: 67.2%) (Figure 3B, Supplementary Table 1). The mean atorvastatin or atorvastatin‐equivalent doses were 18.8 mg/day at admission (LLT group), 22 mg/day during the hospital stay, 21.7 mg/day at discharge, and 21 mg/day at 6‐month follow‐up (Figure 3C, Supplementary Table 2). At admission, of the 214 patients receiving LLT, only 5 (2.3%) were on atorvastatin‐equivalent dose of 40 mg/day and no patient was on high‐dose statins (i.e., atorvastatin or atorvastatin‐equivalent dose >40 mg/day). At discharge in all patients (104/903 [11.5%]) (LLT: 17/209 [8.1%], non‐LLT: 85/687 [12.4%]) and at 6 months follow‐up (73/873 [8.4%]) (LLT: 9/203 [4.4%], non‐LLT: 64/665 [9.6%]) patients were prescribed with atorvastatin‐equivalent dose of 40 mg/day. Surprisingly, no patient was on high‐dose statin at any time‐point (i.e., atorvastatin‐equivalent dose >40 mg/day).

### 3.5 Predictors for Attaining LDL‐C < 1.8 mmol/L 6 months post‐ACS

Overall, 310/752 (41.2%) patients attained LDL‐C level < 1.8 mmol/L at 6 months post‐ACS. Being male (odds ratio [OR] 1.7, 95% CI 1.1 to 2.6, p = 0.009) and undergoing PCI during index hospitalization (OR 1.5, 95% CI 1.1 to 2.1, p = 0.008) were found to be independent positive predictors for LDL‐C goal attainment. Having a history of symptomatic CHF (New York Heart Disease Association II‐IV) negatively predicted LDL‐C goal attainment (OR 0.5, 95% CI, 0.2 to 0.969, *p* = 0.041, Table 2).

**TABLE 2:** Predictors of attaining LDL‐C < 1.8 mmol/L 6 months post‐ACS

Characteristic	Odds Ratio (95% Confidence Interval)
Age (years) (≥65)	1.0 (0.7–1.4)
Gender (male)	1.7 (1.1–2.6)**
BMI (kg/m^2^) (≥28)	1.0 (0.7–1.5)
Statin dose during 6‐month follow‐up (<40 mg/day atorvastatin dose equivalent)	0.7 (0.4 4–1.2)
Hypertension	1.4 (1.0–1.9)
T2DM	1.1 (0.8–1.6)
History of stroke	1.5 (0.9–2.5)
History of symptomatic CHF (NYHA II‐IV)	0.5 (0.2–0.969)*
Sedentary lifestyle	0.9 (0.6–1.2)
Current smoker	0.7 (0.5–1.1)
Further therapy during hospitalization ‐ PCI	1.5 (1.1–2.1)**

*Note*: *P < .05, **P < .01.

Abbreviations: ACS, acute coronary syndrome; BMI, body mass index; CHF, chronic heart failure; NYHA, New York Heart Disease Association; PCI, percutaneous coronary intervention; T2DM, type 2 diabetes mellitus.

## 4 DISCUSSION

The results from this real‐world DYSIS II study in post‐ACS patients from China suggest that less than one‐fifth (17.1%) of patients were at LDL‐C goal of <1.8 mmol/L (per 2016 Chinese Adult Lipid Management Guideline^14^) at ACS occurrence; the percentage of patients achieving the LDL‐C goal increased to 41.2% at 6‐month follow‐up. Our study confirms the finding of poor LDL‐C control observed in the previous DYSIS and DYSIS II cohorts^17^, ^18^, ^19^, ^20^, ^21^ and the recent real‐word studies from China and Italy.^22^, ^23^

A cross‐sectional DYSIS‐China study, which reported a much higher LDL‐C goal attainment rate of 61.5%, had a less stringent LDL‐C goal cut‐off (<2.0 mmol/L) and included non‐ACS patients.^24^ The 6‐month LDL‐C goal attainment rates observed in our study are marginally higher than the rates observed for secondary prevention cohorts of the DYSIS‐China study (33.1%)^25^ and the multinational DYSIS II study excluding China (37.0% at follow‐up).^17^

A considerable proportion of our study patients, having a pre‐admission risk stratification of very‐high and high‐CV risk treated with LLT, were not at their recommended LDL‐C goal (67.6% and 40%), and thus may have been predisposed to ACS. The ICLPS made a similar observation with only 32.1% of the very‐high risk patients achieving their LDL‐C goals (when on LLT ≥3 months) followed by 51.9% of the high‐risk patients.^26^ Although the mean distance to achieve the LDL‐C goal reduced over 6 months post‐ACS, it was still short of 0.7 mmol/L (from 1.8 mmol/L). Even though from pre‐ESC/EAS 2019 guidelines era, these findings reveal gaps between the regional guideline recommendations^14^ and clinical practice on appropriate dose and LLT selection in China. This is evident from inadequacy of the prescribed LLT monotherapy at admission with mean atorvastatin‐equivalent dose of 18.8 mg/day, and especially in the LLT group at discharge (with mean atorvastatin‐equivalent dose of 20.8 ± 6.1 mg/day). Furthermore, statin monotherapy predominated at 6‐month follow‐up as well, in both LLT and non‐LLT groups with a mean atorvastatin‐equivalent dose of 21 ± 6.2 mg/day suggesting a moderate dose. In the previous China‐DYSIS study, the use of statin monotherapy was predominant.^20^ Similar results from our study conducted around 5 years later indicate existence of non‐concurrence to treatment intensification. Even if the dosing used in the present study was compliant with the 2016 Chinese guideline recommendations, a very small percentage of patients (0.9% at admission and < 8% at discharge and 6‐month follow‐up) received combination therapy (statin + ezetimibe). This is in spite of Chinese guidelines recommending use of combination therapy if goals are not reached and also intensification according to patient tolerance.^14^ In the IMPROVE‐IT study, a combination of simvastatin and ezetimibe lowered LDL‐C by approximately 24% and significantly lowered the risk of CV events compared with statin monotherapy in post‐ACS patients.^12^ Lower use of combination therapy could be a reason for the lower rate of LDL‐C goal attainment in our study population. We observed that the rate of LDL‐C goal attainment strikingly improved in the non‐LLT cohort (13.0%–42.4%) at 6 months whereas it did not increase substantially in the LLT group (31.5%–36%) suggesting treatment fatigue. It was observed that the daily atorvastatin‐equivalent statin dose for the LLT group increased from 18.8 mg/dl at admission to 20.8 mg/dl at discharge and then remained approximately at the same dose level for 6 months; whereas for the non‐LLT group, a moderate dose level of mean atorvastatin‐equivalent dose was continued from hospital stay until 6‐month follow‐up. Less than 10% of patients in the non‐LLT group and only 4.4% of patients in the LLT group were on atorvastatin‐equivalent dose of 40 mg/day at 6‐month follow‐up in spite of lower LDL‐C goal achievement rate. In this study, the LLT initiation at moderate level was in line with the Chinese guidelines; however, there was a lack of intensification even at 6 months. Several meta‐analyses have demonstrated that high‐intensity statins, now endorsed by ESC/EAS 2019, avert adverse CV outcomes in post‐ACS patients.^27^, ^28^, ^29^ Though local clinical practice is governed by the regional guidelines, in this case, we found gaps between practice and the latest ESC/EAS guidelines recommending an LDL‐C reduction of ≥50% from baseline, an LDL‐C goal of <1.4 mmol/L (<55 mg/dl), monitoring at 4–6 weeks, and prescribing the highest tolerated statin dose or combinations for secondary prevention in very high‐risk patients.^15^ Thus, rigorous follow‐ups for close monitoring carry immense value.

The possible reasons behind this practice gap may be specific to Asian countries. Even though the efficacy of high‐intensity statins in secondary prevention of adverse CV outcomes is established, a recent study in Chinese patients reported a low LDL‐C goal attainment rate of 36.2% in ACS patients treated with intensive statin therapy.^30^ CHILLAS study found that for ACS patients with a relatively low baseline LDL‐C level receiving optimized current medication and interventional therapy, LDL‐C reduction by 6.4% achieved by double‐dose statins did not bring significant clinical effectiveness.^31^ Moreover, a recent real‐world study in Singapore found that initiation of low‐, medium‐, and high‐intensity statins resulted in a lowering of LDL‐C by similar proportions of 21.6%, 28.9%, and 25.2%, respectively, in the primary care.^32^ These results may stem the belief that Asian population may respond better to low‐to‐moderate dose statins.

The patient and physician related factors for lower LDL‐C goal attainment in a community based study in the US and CEPHEUS II study for secondary prevention were suboptimal adherence, a lower rate of high‐intensity statin prescriptions, dissatisfaction with the treatment, physicians not setting the lipid goals, and guideline nonconcordance.^33^, ^34^ In our study, 88.9% from the LLT group and 81.9% from the non‐LLT group completed 6‐month follow‐up, with lost to follow‐up and withdrawal being the common reasons for discontinuing the study. In all, 20 deaths were also reported. Nonadherence could be the possible reasons for loss to follow‐up. The reasons of nonintensification and not prescribing combination therapy need to be systematically investigated in China. Another patient and physician related factor affecting nonprescription of high‐intensity statins might be cost; however, as most statins have insurance coverage in China, it may not be the factor limiting their use.

Summing up of all these findings suggest that intensification of statin dose is necessary; a rigorous follow‐up may facilitate better monitoring, and patient‐provider interactions to dispel the myths and motivate people for achieving the LDL‐C goal.

We found that the patients having symptomatic CHF were less likely to attain the LDL‐C goal. The DYSIS II‐Europe study has also reported CHF as a negative predictor.^18^ Similar to our findings, male gender, and history of PCI were found to be predictive of LDL‐C goal attainment by Zhang et al. Higher compliance to treatment due to active follow‐up and treatment augmentation after PCI could be possible reasons.

### 4.1 Limitations

The study enrolled patients from tertiary hospitals; hence, the applicability of results to general clinical practice may be limited. In this study, the lipid profile was measured within 24 hours of hospital admission. The LDL‐C goal attainment rates at admission (i.e., at ACS occurrence) may not reflect the chronic LDL‐C levels observed in routine clinical practice in China as LDL‐C decreases during the first day post‐ACS and hence should be interpreted cautiously. Adherence to LLT, an important predictor, was not systematically explored in this study. Secondly, LDL‐C goal attainment was not explored as per monotherapy and combination therapy because the number of patients receiving combination therapy was very small. Around 80.0% of patients returned for follow‐up at 6 months; those who were lost to follow‐up may have been less compliant with their LLT, which remains unexplored. Moreover, a 6‐month follow‐up may not be sufficient to provide information and estimates about the long‐term lipid management in patients with ACS.

## 5 CONCLUSIONS

This real‐world study in China reports a low LDL‐C goal attainment rate in patients at ACS occurrence and 6 months after discharge. The majority of the study patients were on LLT at discharge and 6‐month follow‐up; moderate intensity statin monotherapy was the predominant LLT; only a small proportion of patients were on statin + ezetimibe combination. The study results provide insights into multi‐factorial challenges faced for LDL‐C goal attainment post‐ACS. Inadequate intensification of statin therapy, not introducing combination therapy at the right time, and lack of rigorous follow‐up at 4–6 weeks could be the possible factors contributing to suboptimal management of dyslipidemia. At policy level, collaborative efforts are needed to synchronize regional and international guidelines to prevent disparities in practice by the providers. Sensitization of providers is needed to eliminate clinical inertia and bring in fundamental shifts from the practice of prescribing low‐to‐moderate intensity statins.

## CONFLICT OF INTEREST

Anhua Mao, Rui Bian, Wenmin Tang and Yuqin Ran are employees of MSD China. Other authors have none to declare.

## AUTHORS' CONTRIBUTIONS

Gang Liu, Yujie Zhou, Anhua Mao, Yuqin Ran, and Yong Huo conceived, designed, or planned the study. Yanjun Gong, Xuan Li, Xiang Ma, Hongwei Yu, Ying Li, Haiyan Meng, Jiyan Chen, Guochun Zhang, Bin Wang, Xiaoyong Qi, Xiaofeng Wang, Jianjun Mu, Xitian Hu, Jingping Wang, Shaowen Liu, Gang Liu, Zhenyu Yang, Yujie Zhou, Xiangqing Kong, Yuhu Yan, Changqian Wang, Jian'An Wang, Lijun Wang, Guosheng Fu, Lin Wei, Daoquan Peng, Shuyang Zhang, Ruogu Li, Rui Bian, and Jie Jiang collected or assembled the data. Yujie Zhou provisioned study materials or patients. Gang Liu and WT performed or supervised analyses. Yanjun Gong and Yong Huo drafted the manuscript. Anhua Mao obtained funding. All authors provided substantive suggestions for revision or critically reviewed subsequent iterations of the manuscript, gave final approval, and agreed to be accountable for all aspects of work ensuring integrity and accuracy.

## Supporting information


**Appendix S1**: Supporting Information

## Data Availability

The datasets generated and analyzed during the current study are available from the corresponding author upon reasonable request.
