# Supplementary material for: Lipid goal attainment in post‐acute coronary syndrome patients in China: Results from the 6‐month real‐world dyslipidemia international study II
Source: Clin Cardiol. 2021 Oct 15;44(11):1575–85. doi: 10.1002/clc.23725 (PMC8571548; doi:10.1002/clc.23725)
Supplement: Supplementary file 1 — Appendix S1: Supporting Information [file CLC-44-1575-s001.doc]

Lipid Goal Attainment in Post-acute Coronary Syndrome Patients in China: Results From the 6‑month Real-world Dyslipidaemia International Study II (DYSIS II)

**SUPPLEMENTARY DATA**

**Supplementary Table 1:** Lipid-Lowering Therapy Utilization Patterns at Admission, Hospitalization, Discharge and 6-month Follow up

|  | All Patients | | | LLT Patients | | | Non-LLT Patients | | |
| --- | --- | --- | --- | --- | --- | --- | --- | --- | --- |
|  | (N=1103) | | | (N=216) | | | (N=858) | | |
|  | n | (%) | (95% CI)^†^ | n | (%) | (95% CI)^†^ | n | (%) | (95% CI)^†^ |
| LLT Therapy |  |  |  |  |  |  |  |  |  |
| Admission |  |  |  | 216 |  |  |  |  |  |
| Monotherapy |  |  |  | 214 | (99.1) | (96.7,  99.7) |  |  |  |
| Statins Monotherapy |  |  |  | 213 | (98.6) | (96.0,  99.5) |  |  |  |
| Atorvastatin |  |  |  | 144 | (66.7) | (60.1,  72.6) |  |  |  |
| Simvastatin |  |  |  | 10 | (4.6) | (2.5,  8.3) |  |  |  |
| Rosuvastatin |  |  |  | 51 | (23.6) | (18.4,  29.7) |  |  |  |
| Pravastatin |  |  |  | 1 | (0.5) | (0.1,  2.6) |  |  |  |
| Other |  |  |  | 7 | (3.2) | (1.6,  6.5) |  |  |  |
| Non-statins Monotherapy |  |  |  | 1 | (0.5) | (0.1,  2.6) |  |  |  |
| Combination Therapy |  |  |  | 2 | (0.9) | (0.3,  3.3) |  |  |  |
| Statins + Ezetimibe |  |  |  | 2 | (0.9) | (0.3,  3.3) |  |  |  |
| Statins + Other Non-statins |  |  |  | 0 | (0.0) | (0.0,  1.7) |  |  |  |
| Discharge | 909 |  |  | 210 |  |  | 690 |  |  |
| Monotherapy | 841 | (92.5) | (90.6,  94.1) | 190 | (90.5) | (85.7,  93.8) | 643 | (93.2) | (91.1,  94.8) |

|  | All Patients | | | LLT Patients | | | Non-LLT Patients | | |
| --- | --- | --- | --- | --- | --- | --- | --- | --- | --- |
|  | (N=1103) | | | (N=216) | | | (N=858) | | |
|  | n | (%) | (95% CI)^†^ | n | (%) | (95% CI)^†^ | n | (%) | (95% CI)^†^ |
| Statins Monotherapy | 836 | (92.0) | (90.0,  93.6) | 190 | (90.5) | (85.7,  93.8) | 640 | (92.8) | (90.6,  94.5) |
| Atorvastatin | 597 | (65.7) | (62.5,  68.7) | 127 | (60.5) | (53.7,  66.8) | 466 | (67.5) | (64.0,  70.9) |
| Simvastatin | 3 | (0.3) | (0.1,  1.0) | 2 | (1.0) | (0.3,  3.4) | 1 | (0.1) | (0.0,  0.8) |
| Rosuvastatin | 215 | (23.7) | (21.0,  26.5) | 55 | (26.2) | (20.7,  32.5) | 158 | (22.9) | (19.9,  26.2) |
| Pravastatin | 1 | (0.1) | (0.0,  0.6) | 0 | (0.0) | (0.0,  1.8) | 1 | (0.1) | (0.0,  0.8) |
| Other | 20 | (2.2) | (1.4,  3.4) | 6 | (2.9) | (1.3,  6.1) | 14 | (2.0) | (1.2,  3.4) |
| Non-statins Monotherapy | 5 | (0.6) | (0.2,  1.3) | 0 | (0.0) | (0.0,  1.8) | 3 | (0.4) | (0.1,  1.3) |
| Combination Therapy | 68 | (7.5) | (5.9,  9.4) | 20 | (9.5) | (6.2,  14.3) | 47 | (6.8) | (5.2,  8.9) |
| Statins + Ezetimibe | 66 | (7.3) | (5.7,  9.1) | 19 | (9.0) | (5.9,  13.7) | 46 | (6.7) | (5.0,  8.8) |
| Statins + Other Non-statins | 2 | (0.2) | (0.1,  0.8) | 1 | (0.5) | (0.1,  2.6) | 1 | (0.1) | (0.0,  0.8) |
| 6-month Follow up | 881 |  |  | 204 |  |  | 670 |  |  |
| Monotherapy | 812 | (92.2) | (90.2,  93.8) | 183 | (89.7) | (84.8,  93.2) | 622 | (92.8) | (90.6,  94.6) |
| Statins Monotherapy | 805 | (91.4) | (89.3,  93.1) | 183 | (89.7) | (84.8,  93.2) | 617 | (92.1) | (89.8,  93.9) |
| Atorvastatin | 576 | (65.4) | (62.2,  68.4) | 122 | (59.8) | (53.0,  66.3) | 450 | (67.2) | (63.5,  70.6) |
| Simvastatin | 4 | (0.5) | (0.2,  1.2) | 2 | (1.0) | (0.3,  3.5) | 2 | (0.3) | (0.1,  1.1) |

|  | All Patients | | | LLT Patients | | | Non-LLT Patients | | |
| --- | --- | --- | --- | --- | --- | --- | --- | --- | --- |
|  | (N=1103) | | | (N=216) | | | (N=858) | | |
|  | n | (%) | (95% CI)^†^ | n | (%) | (95% CI)^†^ | n | (%) | (95% CI)^†^ |
| Rosuvastatin | 204 | (23.2) | (20.5,  26.1) | 52 | (25.5) | (20.0,  31.9) | 151 | (22.5) | (19.5,  25.9) |
| Pravastatin | 2 | (0.2) | (0.1,  0.8) | 1 | (0.5) | (0.1,  2.7) | 1 | (0.1) | (0.0,  0.8) |
| Other | 19 | (2.2) | (1.4,  3.3) | 6 | (2.9) | (1.4,  6.3) | 13 | (1.9) | (1.1,  3.3) |
| Non-statins Monotherapy | 7 | (0.8) | (0.4,  1.6) | 0 | (0.0) | (0.0,  1.8) | 5 | (0.7) | (0.3,  1.7) |
| Combination Therapy | 69 | (7.8) | (6.2,  9.8) | 21 | (10.3) | (6.8,  15.2) | 48 | (7.2) | (5.4,  9.4) |
| Statins + Ezetimibe | 68 | (7.7) | (6.1,  9.7) | 20 | (9.8) | (6.4,  14.7) | 48 | (7.2) | (5.4,  9.4) |
| Statins + Other Non-statins | 1 | (0.1) | (0.0,  0.6) | 1 | (0.5) | (0.1,  2.7) | 0 | (0.0) | (0.0,  0.6) |
|  |  |  |  |  |  |  |  |  |  |

**Supplementary Table 2:** Atorvaststin-equivalent Dose at Admission, Hospitalization, Discharge and 6-month Follow up

|  | All Patients | | | LLT Patients | | | Non-LLT Patients | | |
| --- | --- | --- | --- | --- | --- | --- | --- | --- | --- |
|  | (N=1103) | | | (N=216) | | | (N=858) | | |
|  | n | (%) | (95% CI)^†^ | n | (%) | (95% CI)^†^ | n | (%) | (95% CI)^†^ |
| Statin Dose |  |  |  |  |  |  |  |  |  |
| Admission |  |  |  | 214 |  |  |  |  |  |
| Atorvastatin Dose equiv. Mean±SD |  |  |  | 18.8±5.1 |  |  |  |  |  |
|  |  |  |  |  |  |  |  |  |  |
| <40 mg/day Atorvastatin Dose Equiv. |  |  |  | 209 | (97.7) | (94.6,  99.0) |  |  |  |
| 5 mg/day Atorvastatin Dose Equiv. |  |  |  | 5 | (2.3) | (1.0,  5.4) |  |  |  |
| 10 mg/day Atorvastatin Dose Equiv. |  |  |  | 28 | (13.1) | (9.2,  18.3) |  |  |  |
| 20 mg/day Atorvastatin Dose Equiv. |  |  |  | 180 | (84.1) | (78.6,  88.4) |  |  |  |
| ≥40 mg/day Atorvastatin Dose Equiv. |  |  |  | 5 | (2.3) | (1.0,  5.4) |  |  |  |
| 40 mg/day Atorvastatin Dose Equiv. |  |  |  | 5 | (2.3) | (1.0,  5.4) |  |  |  |
| >40 mg/day Atorvastatin Dose Equiv. |  |  |  | 0 | (0.0) | (0.0,  1.8) |  |  |  |
|  |  |  |  |  |  |  |  |  |  |
| During Hospital Stay | 846 |  |  | 207 |  |  | 631 |  |  |
| Atorvastatin Dose equiv. Mean±SD | 22.0±7.6 |  |  | 20.3±6.7 |  |  | 22.7±7.7 |  |  |
|  |  |  |  |  |  |  |  |  |  |
| <40 mg/day Atorvastatin Dose Equiv. | 725 | (85.7) | (83.2,  87.9) | 188 | (90.8) | (86.1,  94.0) | 531 | (84.2) | (81.1,  86.8) |
| 5 mg/day Atorvastatin Dose Equiv. | 4 | (0.5) | (0.2,  1.2) | 3 | (1.4) | (0.5,  4.2) | 1 | (0.2) | (0.0,  0.9) |
| 10 mg/day Atorvastatin Dose Equiv. | 56 | (6.6) | (5.1,  8.5) | 29 | (14.0) | (9.9,  19.4) | 26 | (4.1) | (2.8,  6.0) |
| 20 mg/day Atorvastatin Dose Equiv. | 718 | (84.9) | (82.3,  87.1) | 191 | (92.3) | (87.8,  95.2) | 521 | (82.6) | (79.4,  85.3) |
| ≥40 mg/day Atorvastatin Dose Equiv. | 121 | (14.3) | (12.1,  16.8) | 19 | (9.2) | (6.0,  13.9) | 100 | (15.8) | (13.2,  18.9) |
| 40 mg/day Atorvastatin Dose Equiv. | 121 | (14.3) | (12.1,  16.8) | 19 | (9.2) | (6.0,  13.9) | 100 | (15.8) | (13.2,  18.9) |
| >40 mg/day Atorvastatin Dose Equiv. | 1 | (0.1) | (0.0,  0.7) | 0 | (0.0) | (0.0,  1.8) | 1 | (0.2) | (0.0,  0.9) |
|  |  |  |  |  |  |  |  |  |  |
| Discharge | 903 |  |  | 209 |  |  | 687 |  |  |
|  |  |  |  |  |  |  |  |  |  |
| Atorvastatin Dose equiv. Mean±SD | 21.7±6.8 |  |  | 20.8±6.1 |  |  | 22.0±7.0 |  |  |
|  |  |  |  |  |  |  |  |  |  |
| <40 mg/day Atorvastatin Dose Equiv. | 799 | (88.5) | (86.2,  90.4) | 192 | (91.9) | (87.4,  94.9) | 602 | (87.6) | (85.0,  89.9) |
| 5 mg/day Atorvastatin Dose Equiv. | 1 | (0.1) | (0.0,  0.6) | 0 | (0.0) | (0.0,  1.8) | 1 | (0.1) | (0.0,  0.8) |
| 10 mg/day Atorvastatin Dose Equiv. | 45 | (5.0) | (3.7,  6.6) | 16 | (7.7) | (4.8,  12.1) | 28 | (4.1) | (2.8,  5.8) |
| 20 mg/day Atorvastatin Dose Equiv. | 761 | (84.3) | (81.8,  86.5) | 182 | (87.1) | (81.9,  91.0) | 574 | (83.6) | (80.6,  86.1) |
| ≥40 mg/day Atorvastatin Dose Equiv. | 104 | (11.5) | (9.6,  13.8) | 17 | (8.1) | (5.1,  12.6) | 85 | (12.4) | (10.1,  15.0) |
| 40 mg/day Atorvastatin Dose Equiv. | 104 | (11.5) | (9.6,  13.8) | 17 | (8.1) | (5.1,  12.6) | 85 | (12.4) | (10.1,  15.0) |
| >40 mg/day Atorvastatin Dose Equiv. | 0 | (0.0) | (0.0,  0.4) | 0 | (0.0) | (0.0,  1.8) | 0 | (0.0) | (0.0,  0.6) |
|  |  |  |  |  |  |  |  |  |  |
| 6-month Follow up | 873 |  |  | 203 |  |  | 665 |  |  |
| Atorvastatin Dose equiv. Mean±SD | 21.0±6.2 |  |  | 20.2±4.9 |  |  | 21.3±6.5 |  |  |
|  |  |  |  |  |  |  |  |  |  |
| <40 mg/day Atorvastatin Dose Equiv. | 800 | (91.6) | (89.6,  93.3) | 194 | (95.6) | (91.8,  97.7) | 601 | (90.4) | (87.9,  92.4) |
| 5 mg/day Atorvastatin Dose Equiv. | 4 | (0.5) | (0.2,  1.2) | 0 | (0.0) | (0.0,  1.9) | 4 | (0.6) | (0.2,  1.5) |
| 10 mg/day Atorvastatin Dose Equiv. | 49 | (5.6) | (4.3,  7.3) | 15 | (7.4) | (4.5,  11.8) | 33 | (5.0) | (3.6,  6.9) |
| 20 mg/day Atorvastatin Dose Equiv. | 753 | (86.3) | (83.8,  88.4) | 182 | (89.7) | (84.7,  93.1) | 567 | (85.3) | (82.4,  87.8) |
| ≥40 mg/day Atorvastatin Dose Equiv. | 73 | (8.4) | (6.7,  10.4) | 9 | (4.4) | (2.3,  8.2) | 64 | (9.6) | (7.6,  12.1) |
| 40 mg/day Atorvastatin Dose Equiv. | 73 | (8.4) | (6.7,  10.4) | 9 | (4.4) | (2.3,  8.2) | 64 | (9.6) | (7.6,  12.1) |
| >40 mg/day Atorvastatin Dose Equiv. | 0 | (0.0) | (0.0,  0.4) | 0 | (0.0) | (0.0,  1.9) | 0 | (0.0) | (0.0,  0.6) |
| Medications taken before the day prior to admission are considered as medications at admission. Medications taken from the day prior to admission (inclusive) to the day of discharge (exclusive) are considered as medications during hospital stay. Medications taken over the day of discharge are considered as medications at discharge. Medications taken over the day of 6-month follow up are considered as medications at 6-month follow up. One medication may be classified into multiple time points.  Percentages are based on the number of patients with valid data at each time point.  ^†^ Based on Wilson score method. | | | | | | | | | |
